# Improving Antibiotic Activity against Wound Pathogens with Manuka Honey *In Vitro*

**DOI:** 10.1371/journal.pone.0045600

**Published:** 2012-09-26

**Authors:** Rowena Jenkins, Rose Cooper

**Affiliations:** Centre for Biomedical Sciences, Cardiff School of Health Sciences, Cardiff Metropolitan University, Cardiff, United Kingdom; University of Malaya, Malaysia

## Abstract

Following the discovery of synergistic action between oxacillin and manuka honey against methicillin-resistant *Staphylococcus aureus*, this study was undertaken to search for further synergistic combinations of antibiotics and honey that might have potential in treating wounds. Fifteen antibiotics were tested with and without sublethal concentrations of manuka honey against each of MRSA and *Pseudomonas aeruginosa* using disc diffusion, broth dilution, E strip, chequerboard titration and growth curves. Five novel antibiotic and manuka honey combinations were found that improved antibacterial effectiveness *in vitro* and these offer a new avenue of future topical treatments for wound infections caused by these two important pathogens.

## Introduction

Methicillin-resistant *Staphylococcus aureus* (MRSA) and *Pseudomonas aeruginosa* (*P. aeruginosa*) are important nosocomial pathogens that cause serious infections with significant associated morbidity and mortality, especially in people with weakened immune systems. Both have been implicated in wound infection and management of infections has been complicated by the emergence and continued prevalence of multiple drug resistant (MDR) strains with resistance determinants to antibiotics of several different classes [1], [2]. The problem of finding effective treatments has been delayed by reduced investment in the discovery and development of new antibiotics.

Substantial efforts to find effective therapies by combining different antibiotics have been made in the treatment of MDR infections such as tuberculosis. Combination therapy has been promoted as a strategy for reducing the emergence of antibiotic resistant strains because the use of two or more antimicrobials with differing modes of action decreases the likelihood of an organism possessing the traits necessary to survive [3]. An advantage of combination therapy is that less of each antimicrobial agent needs to be administered, thus reducing treatment costs and the possibility of side effects. Using synergistic combinations of antimicrobial agents provides a means to achieve greater efficacy than that expected by combining the two non-synergistic agents.

Natural products that have shown potential for future use with current antibiotics include gallocatechins, *Saliva miltiorrhiza* and curcuminoids [4]–[6]. Honey is another possible candidate for synergistic action with antibiotics. The first report of synergistic action between an Indian honey and antibiotics against MDR bacteria isolated from clinical specimens was in 1998 [7]. Combinations of honey and each of gentamicin, amikacin and ceftazidime tested by broth dilution were shown to act synergistically in inhibiting six strains of *P. aeruginosa* but not eight *Klebsiella* strains. One Omani honey selected from 30 samples was shown to enhance the activity of gentamicin against *Staphylococcus aureus* by 22% within 30 minutes [8]. Neither of these studies deduced numerical values to support their deductions of synergy.

Although an ancient topical treatment for wounds, honey has been re-accepted into conventional medicine and it is currently available as a licensed medical device either incorporated into sterile dressings or sterilised in tubes. Manuka honey is one of the medical grade honeys available that is used in these formulations. It has been shown to inhibit the growth of many organisms *in vitro* including *S. aureus* [9] and *P. aeruginosa* [10] and can eradicate bacteria from colonised wounds [11]. Insights into the mode of action of honey for *S. aureus*, *P. aeruginosa* and MRSA have been made [12]–[14].

**Table 1:** Susceptibility of EMRSA-15 to honey and antibiotics alone and in combination.

Antibiotic	Test method	MIC (µg/ml) antibiotic		MIC (% w/v) MH*		FIC antibiotic	FIC MH	FICI
		alone	with MH*	alone	With antibiotic			
Rifampicin	Broth dilution	0.0156	0.0156	6	NT	1	–	–
	Chequerboard	NT	NT	NT	NT	–	–	–
	E strip	0.004	<0.002	NT	NT	<0.5	–	–
	Growth curve	NT	NT	NT	NT	NT	–	–
Tetracycline	Broth dilution	0.5	0.0312	6	NT	<0.5	–	–
	Chequerboard	1.0	0.125	6	2	0.125	0.33	<0.5
	E strip	1.0	0.0312	NT	NT	<0.5	–	–
	Growth curve	0.5	0.125	6	NT	<0.5	–	–
Imipenem	Broth dilution	16	.05	6	NT	<0.5	–	–
	Chequerboard	8	0.0625	6	3	0.0078	0.5	0.5
	E strip	4	0.0312	NT	NT	<0.5	–	–
	Growth curve	8	0.25	6	NT	<0.5	–	–
Mupirocin	Broth dilution	0.06	0.0078	6	NT	<0.5	–	–
	Chequerboard	0.125	0.0078	6	2	0.06	0.33	<0.05
	E strip	<0.06	<0.06	NT	NT	NT	–	–
	Growth curve	0.06	0.0078	6	NT	<0.5	–	–

* MH  =  manuka honey; 5%(w/v) was used in synergy tests.

**Figure 1:**
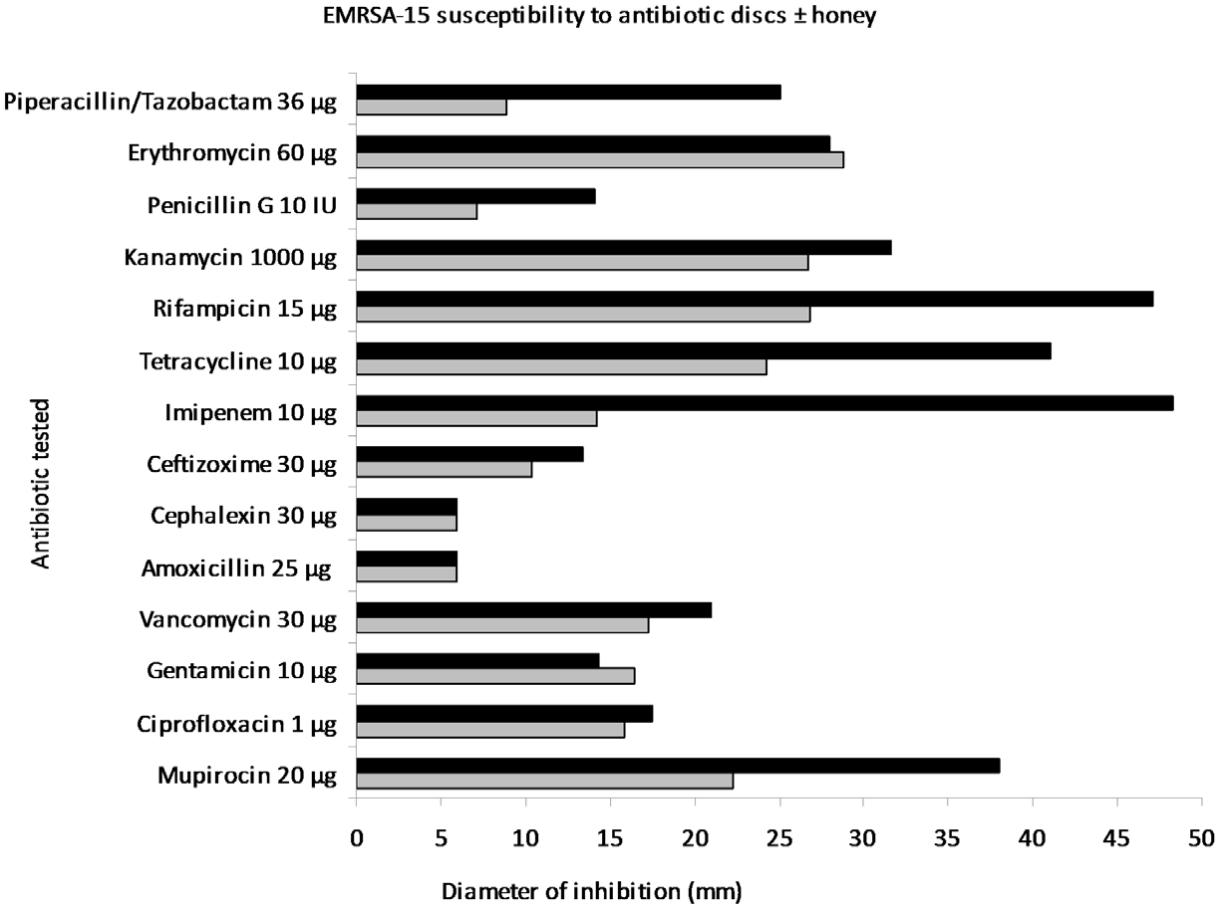
Antibiotic sensitivity testing of EMRSA-15 by disc diffusion. Diameters of zones of inhibition of antibiotics (mm) against EMRSA-15 on plates of Mueller Hinton agar without (grey bars) and with (black bars) 5% (w/v) manuka honey.

**Figure 2:**
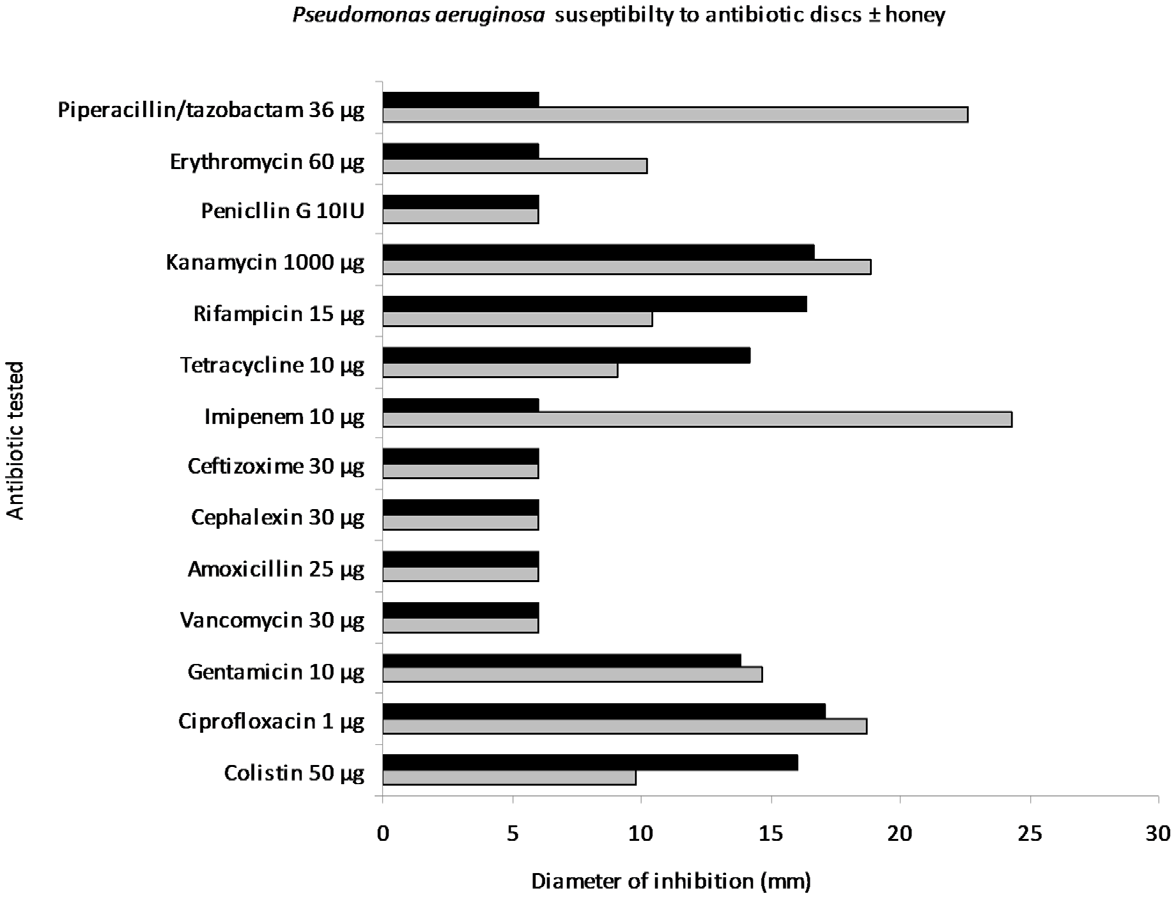
Antibiotic sensitivity testing of *Pseudomonas aeruginosa* by disc diffusion. Diameters of zones of inhibition of antibiotics (mm) against *P.aeruginosa* on plates of Mueller Hinton agar without (grey bars) and with (black bars ) 5% (w/v) manuka honey.

**Figure 3:**
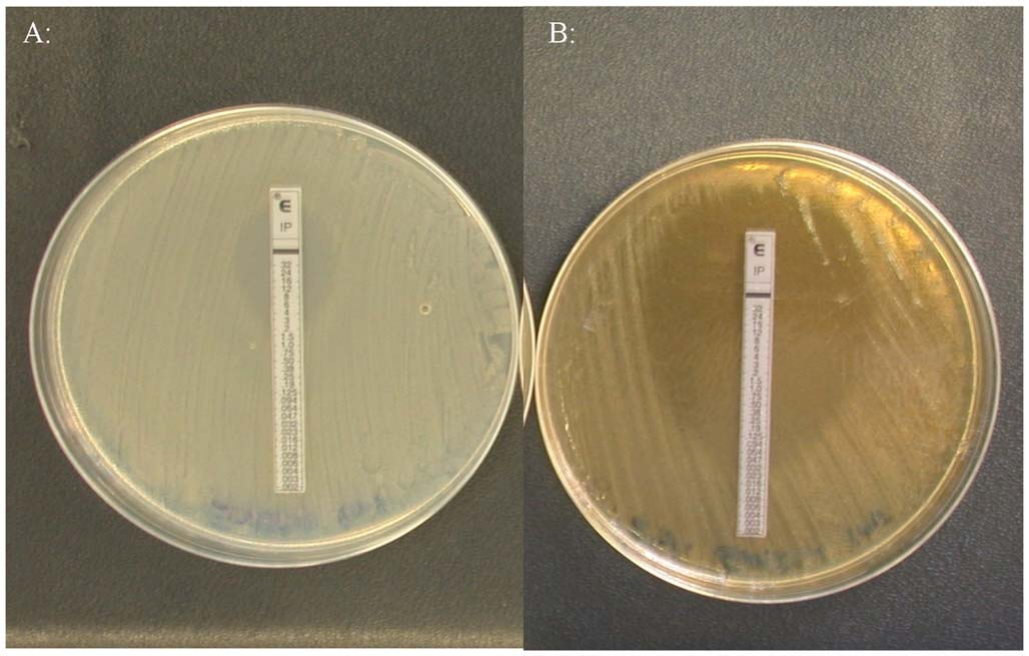
Sensitivity of EMRSA-15 to imipenem by E strip testing. a) MIC of 4 µg/ml imipenem was seen on Mueller Hinton agar. b) MIC of 0.032 µg/ml imipenem was seen on Mueller Hinton agar containing 5% (w/v) manuka honey.

**Figure 4:**
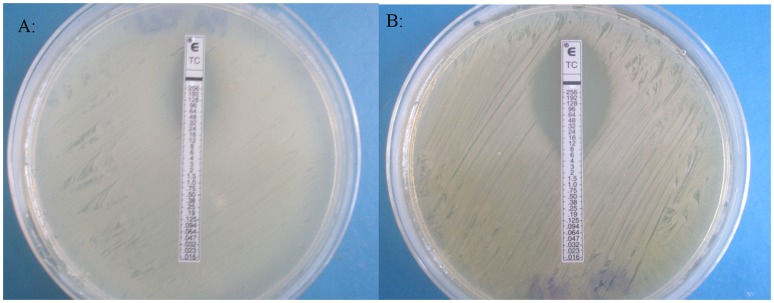
Sensitivity of *Pseudomonas aeruginosa* to tetracycline by E strip testing. a) MIC of 32 µg/ml tetracycline was seen on Mueller Hinton agar. b) MIC of 8 µg/ml tetracycline was seen on Mueller Hinton agar containing 5% (w/v) manuka honey.

Recently synergistic action between piperacillin and methylglyoxal (an antibacterial component characteristically found in manuka honey) was demonstrated by disc diffusion and chequerboard experiments against MDR clinical isolates of *P. aeruginosa* [15]. Synergistic combinations of methylglyoxal with carbenicillin and with amikacin were also noted against *P. aeruginosa*. Furthermore, synergy between oxacillin and manuka honey in the inhibition of MRSA has been reported [16]. Manuka honey, therefore, seems to offer real potential in providing novel synergistic combinations with antibiotics for treating wound infections of MDR bacteria. In this study a selection of antibiotics which affect a wide variety of cellular target sites was tested for synergistic activity with medical grade manuka honey in order to identify novel therapies and five combinations were identified.

## Methods

### Ethics

N/A.

### Bacteria Tested

Epidemic methicillin resistant *Staphylococcus aureus* EMRSA-15 (NCTC 13142) and *Pseudomonas aeruginosa* (NCIMB 8626) were used throughout this study.

### Inhibitors Used in the Study

Fifteen antibiotics were tested: amoxicillin, penicillin G, cephalexin, ceftizoxime, colistin, erythromycin, gentamicin, imipenem, kanamycin, mupirocin, piperacillin/tazobactam, ciprofloxacin, rifampicin, tetracycline and vancomycin. Antibiotic impregnated susceptibility testing discs were purchased from Oxoid (Cambridge, UK) and, except for piperacillin/tazobactam, antibiotics in powdered form were purchased from Sigma (Dorset, UK). Colistin was tested against only *P. aeruginosa* and mupirocin was tested against only MRSA. The manuka honey used in the study was Manukacare 18+ and it was provided by Comvita, UK.

### Antibiotic Sensitivity Testing (AST)

Antibiotic susceptibility was determined using disc diffusion according to the guidelines published by the British Society for Antimicrobial Chemotherapy (BSAC), except that Mueller-Hinton agar (MHA; Oxoid, Cambridge, UK) was used in place of isosensitest agar [17].

### Determination of Minimum Inhibitory Concentration (MIC)

The MIC of each antibiotic was determined by serial doubling dilution with Mueller Hinton Broth (MHB; Oxoid, Cambridge, UK) in microtitre plates. The MIC of manuka honey was also determined in microtitre plates by dilution in MHB, except that dilutions varied by 1% (w/v) intervals, instead of doubling dilutions. Microtitre plates were inoculated with approximately10^5^ cfu/mL and incubated at 37°C for 24 h. The lowest concentration to prevent visible growth was recorded as the MIC. MICs of antibiotics were also determined with E-strips (bioMérieux, Basingstoke, UK) using lawn plates of test bacteria on MHA, according to the manufacturer’s instructions.

### Testing for Synergistic Antibiotic and Honey Combinations by AST

To screen for antibiotic and honey combinations with potential synergistic activity, disc diffusion tests were repeated with MHA containing 5% (w/v) manuka honey. This sub-lethal honey concentration was 1% (w/v) below the MIC of manuka honey. Antibiotics demonstrating increased zones of inhibition were further investigated, but combinations without were not.

### Testing for Synergistic Antibiotic and Honey Combinations by MIC

Similarly MICs of antibiotics were repeated by serial doubling dilution in MHB containing 5% (w/v) manuka honey. MICs were also determined in microtitre plates by chequerboard dilution where serial doubling dilutions of the antibiotic and serial doubling dilutions of manuka honey in MHB were prepared. Microtitre plates were inoculated with approximately10^5^ cfu/mL and incubated at 37°C for 24 h.

### Testing for Synergistic Antibiotic and Honey Combinations by E Strips

The MIC of each antibiotic was confirmed by E strips and MHA containing 5% (w/v) manuka honey.

### Testing for Synergistic Antibiotic and Honey Combinations by Growth Curves

The lowest concentration of an antibiotic needed to prevent the growth of a test organism in the presence and absence of 5% (w/v) manuka honey was determined by monitoring optical density at 550 nm at hourly intervals over 23 h in microtitre plates incubated at 37°C in a Tecan Infinite plate reader. Doubling dilutions of antibiotic were tested in MHB with and without 5% (w/v) manuka honey.

All experiments were performed on three occasions.

For each method used, the fractional inhibition concentration (FIC) was calculated for each antibiotic, where FIC  =  MIC of the antibiotic used in combination with manuka honey divided by its MIC alone. Also FIC of honey was calculated from chequerboard experiments, where FIC  =  MIC of manuka honey used in combination with an antibiotic divided by its MIC alone. Fractional inhibition concentration index (FICI) was calculated for each combination of antibiotic and manuka honey tested by chequerboard. FICI  =  FIC of an antibiotic + FIC of manuka honey. The results were interpreted as follows: ≤0.5– synergy; >0.5 to ≤4– additivity and >4 - antagonism.

**Table 2:** Susceptibility of *Pseudomonas aeruginosa* to honey and antibiotics alone and in combination.

Antibiotic	Test method	MIC (µg/ml) antibiotic		MIC (% w/v) MH*		FIC antibiotic	FIC MH	FICI
		alone	withMH*	alone	Withantibiotic			
Rifampicin	Broth dilution	8	4	6	NT	0.5	–	–
	Chequerboard	8	4	7	3	0.5	0.4	0.9
	E strip	10	3.5	NT	NT	<0.5	–	–
	Growth curve	8	4	6	NT	0.5	–	–
Tetracycline	Broth dilution	32	16	6	NT	0.5	–	–
	Chequerboard	16	8	7	1	0.5	0.1	0.6
	E strip	32	8	NT	NT	<0.5	–	–
	Growth curve	8	4	6	NT	0.5	–	–
Colistin	Broth dilution	2	0.5	6	NT	<0.5	–	–
	Chequerboard	4	2	7	2	0.5	0.3	0.8
	E strip	2	0.4	NT	NT	<0.5	–	–
	Growth curve	2	0.5	6	NT	<0.5	–	–

* MH  =  manuka honey; 5%(w/v) was used in synergy tests.

## Results

The MIC of manuka honey against MRSA was found to be 6% (w/v) in serial broth dilutions, chequerboards and growth curves (Table 1) and 6, 7 and 6% (w/v), respectively, against *P. aeruginosa* (Tables 1 and 2). Therefore 5% (w/v) manuka honey was used in testing for synergistic combinations. Determining antibiotic susceptibility by disc diffusion showed that several antibiotics exhibited increased sensitivity in the presence of manuka honey and allowed for any antibiotics without increased activity to be discarded at this point. Initially the most effective combinations against EMRSA-15 were observed to be piperacillin/tazobactam, rifampicin, tetracycline, imipenem and mupircoin (Fig. 1). Against *P. aeruginosa* rifampicin, tetracycline and colistin showed augmented activity in the presence of honey (Fig. 2). E strips provided a confirmation of these effects (selected examples are shown in Fig. 3 and 4), but the susceptibility of MRSA to rifampicin without honey made it difficult to perceive increased susceptibility in the presence of honey. Also it was decided not to test the piperacillin/tazobactam combination further but to concentrate on single antibiotics.

**Figure 5:**
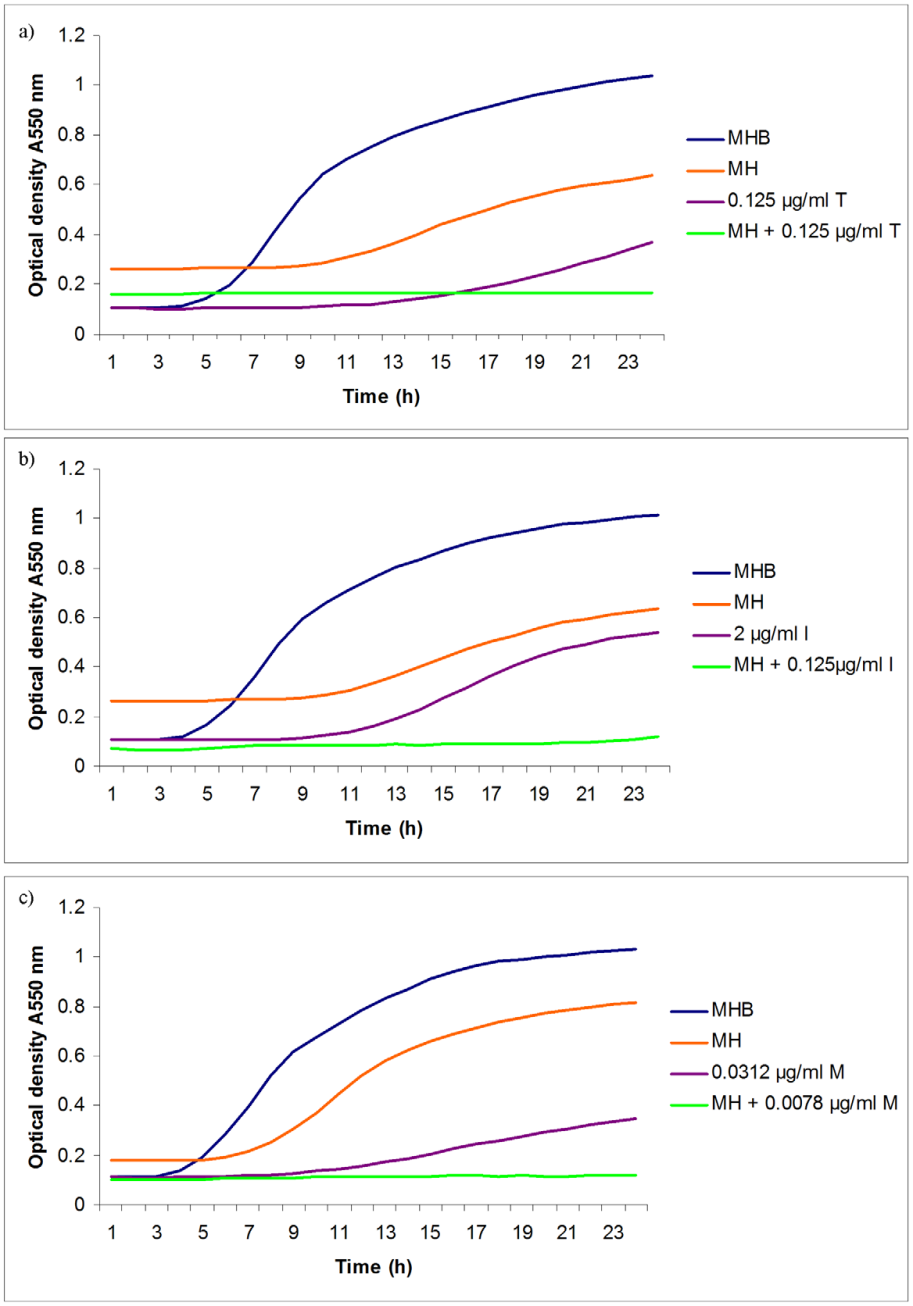
Effect of antibiotics, manuka honey singly and in combination on the growth of EMRSA-15. EMRSA-15 was cultivated in microtitre plates in Mueller Hinton agar with a range of concentrations of antibiotic with and without 5% (w/v) manuka honey and optical density at 550 nm monitored with time. Only the lowest concentration of antibiotic to allow growth is shown in each experiment. MHB = Mueller Hinton broth; MH = MHB with 5% (w/v) manuka honey; in a) T = tetracycline; in b) I = imipenem; in c) M = mupirocin.

**Figure 6:**
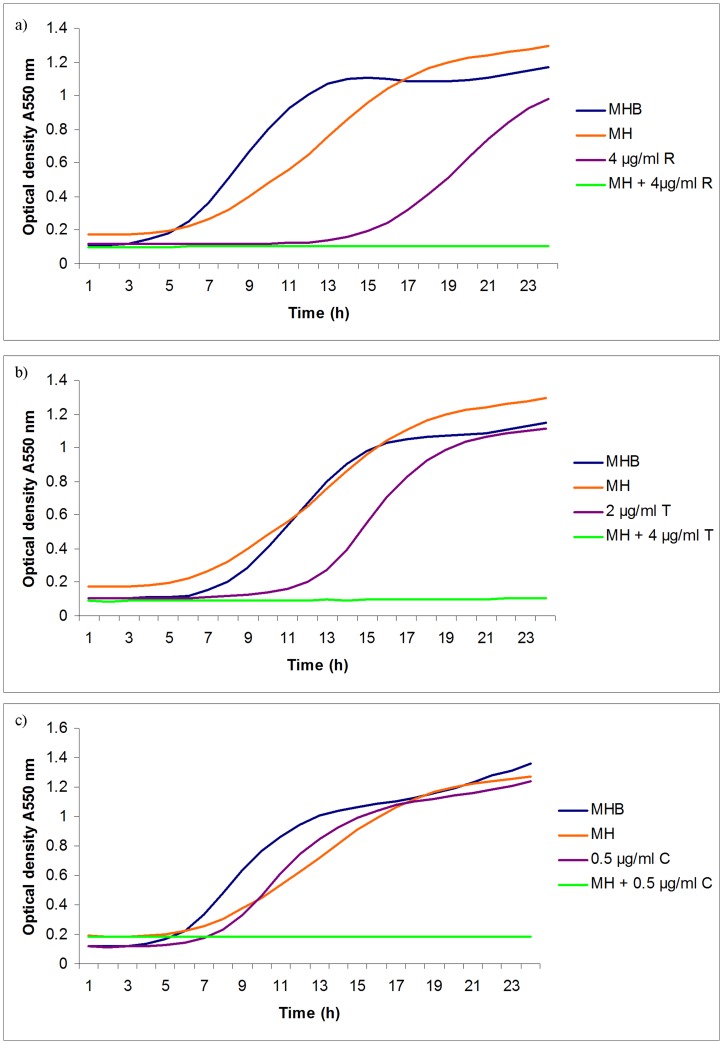
Effect of antibiotics, manuka honey singly and in combination on the growth of *Pseudomonas aeruginosa*. *P. aeruginosa* was cultivated in microtitre plates in Mueller Hinton agar with a range of concentrations of antibiotic with and without 5% (w/v) manuka honey and optical density at 550 nm monitored with time. Only the lowest concentration of antibiotic to allow growth is shown in each experiment. MHB = Mueller Hinton broth; MH = MHB with 5% (w/v) manuka honey; in a) R = rifampicin; in b) T = tetracycline; in c) C = colistin.

Determining MICs by broth dilution with and without manuka honey showed that activity of rifampicin against MRSA was not enhanced and this antibiotic was not investigated further with this bacterium. MICs of the remaining antibiotics determined with and without manuka honey by broth dilution, yielded FICs ≤0.5 in every case (Table 1 and 2), which suggested synergy. These were confirmed by chequerboard experiments and FICIs were calculated to be less than 1.0.

The effect of manuka honey in potentiating antibiotic activity was best illustrated by growth curves where concentrations of antibiotics significantly below their MICs values inhibited growth of test organisms in the presence of sublethal concentrations of manuka honey (Fig. 5 and 6). Synergistic activity between manuka honey and each of tetracycline, imipenem and mupirocin were discovered for MRSA, and additivity between each of rifampicin, tetracycline and colistin and manuka honey for *P. aeruginosa*.

**Table 3:** Antibiotics and their target sites.

Antimicrobial agent	Antimicrobial Class	Target	Reference
Tetracycline	Polyketide	30 S ribosome	26
Rifampicin	Rifamycin	Inhibits DNA-dependent RNA polymerase in bacterial cells by binding tothe beta-subunit, thus preventing transcription to RNA	27
Colistin	Polypeptide	Cell membrane	28
Imipenem	β - lactam	Penicillin-binding proteins	29
Mupirocin	Monoxycarbolic acid	Inhibition of bacterial isoleucyl-tRNA synthetase, blocking RNA and protein synthesis	30

The antibiotics which demonstrated synergistic relationships with manuka honey against either EMRSA-15 or *P. aeruginosa* affect a range of target sites. This indicates that different target sites are affected due to the complex chemistry of honey.

## Discussion

Infections with *Staphylococcus* or *Pseudomonas* species are notoriously difficult to treat as both organisms exhibit resistance to multiple antibiotics, yet few new antibiotics are currently in development [18], [19]. It has been shown that combinations of antibiotics with non antibiotic substances can enhance the efficacy of a number of currently used antibiotics by forming syncretic combinations [20], [21]. Many natural compounds have previously been shown to have potential to inhibit antibiotic resistance in bacteria [22].

In this study, three antibiotics, from an initial selection of fifteen antibiotics proved to be synergistic in combination with sublethal levels of manuka honey against MRSA and three were additive against *P. aeruginosa.* One combination (manuka honey and tetracycline) exhibited enhanced activity against both of the test bacteria, suggesting that it may be the best combination for further investigation. Wounds tend to support diverse polymicrobial communities. Since tetracycline has a broad spectrum of activity, it would be interesting to determine whether synergistic effects are seen with a wider range of wound pathogens.

Several different methods were used in this study to ensure that putative synergistic combinations were confirmed, as it has been noted that different methods may give differing results. This was seen here in the case of rifampicin and honey against MRSA. The initial observations with antibiotic sensitivity discs and E-strips indicated synergy; however this did not translate across to the other tests and broth dilutions gave no change in MICs (Table 1). Although it is worth noting that for all other combinations tested here, the decrease in MIC of antibiotic when combined with manuka honey recorded by broth dilutions was consistent across the other methods used here.

Imipenem and manuka honey were found to be synergistic towards MRSA (Table 1), but not towards *P. aeruginosa* (Fig. 1). Although this observation would exclude this particular combination from being considered for topical use in wounds generally, it is still conceivable that it might play a role in the removal of MRSA from colonised wounds and other body sites. Mupirocin is already used in this context, so improving its efficacy by adding low concentrations of manuka honey is feasible. It may also help to overcome mupirocin resistance in staphylococci and is worthy of continued investigation.

Colistin can be expected to have little effect on Gram positive bacteria and nephrotoxicity limits its systemic use against Gram negative infections. Finding that its activity was enhanced by manuka honey suggests that it might be used topically in treating wounds with persistent Gram negative infections and this observation warrants further research.

Although two research groups have reported synergy between gentamicin and honey [7], [8], this was not replicated here with manuka honey. This could be due to differences in composition of honey. It is likely that the botanical origin of honey influences its biological activity because different antibacterial components have been identified in different honey samples [23]. This makes it important to select an appropriate honey for clinical use.

The use of antibiotics exerts selection pressures that favour the emergence of mutants with antibiotic resistance determinants. Training experiments with manuka honey indicate that bacteria failed to manifest resistance to honey in the laboratory. [24], [25]. It can be postulated that combinations of antibiotic and honey would be less likely to encourage the emergence of MRD bacteria than antibiotics alone.

Modern wound care dressings that contain manuka honey normally use either undiluted honey or at least 80% honey by weight, but the tests employed to demonstrate synergy with antibiotics here employed a sublethal concentration of (5% w/v). Manuka honey is produced in New Zealand and the increased clinical use within the past ten years has called for an increased supply of medical grade manuka honey. With the well publicised bee colony losses globally, it is possible that the supply of manuka honey will not meet clinical demand in the future, so using low concentrations of honey in topical treatments that also contain antibiotics could provide a way of using it most effectively to prolong supply. Since manuka honey is already used in licensed wound dressings and the antibiotics used here are also regulated medical products, regulatory barriers to introducing a new product containing honey and antibiotics might be relatively easily overcome. However changing attitudes to clinical practice might take longer because objective clinical evidence will be required.

It is interesting to note that the antibiotics that have shown synergy with manuka honey in this study are from different antibiotic classes, which inhibit distinct targets such as the 30 S ribosome, RNA polymerase, membranes and penicillin binding proteins (Table 3). This supports the idea that honey is a complex substance perhaps with multiple active components that affect more than one cellular target site.

Further investigation will be needed to confirm whether the combinations identified here are effective against clinical isolates and biofilms. This information will influence whether they are taken forward to be developed for clinical use. There are also other antibiotics that could be tested with manuka honey to discover even more synergistic combinations.
